# Ottawa Ankle Rules: A Reliable Clinical Instrument to Detect Fractures in Children and Adolescents

**DOI:** 10.1055/s-0044-1800938

**Published:** 2025-03-04

**Authors:** Murilo Oliveira de Carvalho, Henrique de Medeiros Marcolino, Marcelo Romancini Daleffe

**Affiliations:** 1 Universidade do Extremo Sul Catarinense, Criciúma, SC, Brasil; 2 Departamento de Ortopedia e Traumatologia, Hospital São José, Criciúma, SC, Brasil

**Keywords:** ankle, foot, pediatrics, radiography, sprains and strains, wounds and injuries

## Abstract

**Objective** To evaluate the accuracy of the Ottawa Ankle Rules (OARs) applied to the medical records of a high-complexity hospital and a private clinic and to analyze their clinical utility in subjects aged 16 or younger.

**Methods** The present is a cross-sectional, analytical observational study with secondary data collected from an orthopedic emergency department and a private orthopedic clinic. The sample consisted of 144 subjects, including all patients with ankle torsion episodes who underwent ankle and/or foot radiographs upon admission. We adopted the OARs as the diagnostic test to calculate the sensitivity, specificity, positive predictive value (PPV), negative predictive value (NPV), and positive and negative likelihood ratios using the radiographic examination finding as the gold standard.

**Results** A total of 191 patients were selected, 144 of whom were included after the application of the inclusion criteria. The sensitivity and NPV of the OARs for fracture identification were of 100%, with the most sensitive test and highest NPV being the inability to walk four steps. We identified a potential reduction of 43.8% in the total number of radiographs requested if only patients with positive OARs underwent the examination.

**Conclusions** The OARs seem to be a reliable clinical tool in ankle sprain management and a useful clinical protocol to exclude fractures in pediatric patients. Their use demonstrated their ability to reduce unnecessary radiograph requests, minimizing radiation exposure and healthcare system costs.

## Introduction

Ankle injuries are common in emergency departments, accounting for 6 to 12% of visits. 1 Ankle fractures account for approximately 5% of all fractures in pediatric patients. 2 The peak incidence of these injuries occurs from 8 to 15 years of age, affecting 2-fold more boys. 3 Most patients with foot and ankle trauma undergo radiography in the emergency department because of potential fractures, although their frequency is lower than 15%. This conservative approach results in many unnecessary requests for radiography, which leads to avoidable radiation exposure, additional costs to the healthcare system, and prolonged waiting times for patients. 4

To standardize diagnostic methods and reduce unnecessary radiography requests, a research group introduced the Ottawa Ankle Rules (OARs) in 1992, which enable the prediction of fracture occurrence in acute ankle injuries through a targeted physical examination. 5 The protocol states that radiographic evaluation is only essential when the patient reports malleolar pain associated with lateral or medial malleolar pain on bone palpation (both 6 cm distal in the posterior region) or inability to support the limb (immediately after the injury and walking four steps in the emergency room). Another indication consists of cases of ankle trauma with referred pain in the midfoot with one or more of the following criteria: pain on bone palpation at the base of the fifth metatarsal, pain on navicular bone palpation, and inability to support the limb (immediately after the injury and walking four steps in the emergency room). 6 At first, radiography alone is required when the clinical criteria are met. 7 Other supplementary tests, such as computed tomography (CT), magnetic resonance imaging (MRI), and blood tests, are reserved for specific situations, such as assessments of joint congruence, intra-articular lesions, and surgical planning. 8 The protocol is contraindicated in patients with altered mental status, drug intoxication, language barriers, or significant edema limiting bone palpation. 6

The sensitivity of the OARs reaches virtually 100% in studies involving the adult population. 9 However, international authors 10 11 12 13 have identified an opportunity to use the OARs in the pediatric population. Application of the OARs to pediatric patients is scarce, possibly due to the challenge of obtaining a reliable verbal history and performing a proper physical examination in some children compared with adults. Furthermore, the OARs can only be applied to children who could walk independently before the episode. 12

The OARs have already been validated in adults, but there is a lack of studies involving the pediatric population regarding the potential use of the rules as a diagnostic screening tool. In the present study, we aimed to evaluate their applicability in pediatric patients treated in a high-complexity hospital and a private clinic.

## Materials and Methods

The present is an observational, cross-sectional, and analytical study with a quantitative approach. The institutional Ethics Committee approved and supervised the project (CAAE: 60851622.1.0000.0119).

The sample included 144 patients aged 16 or younger, treated from January 2019 to December 2022, who underwent radiographs after an episode of ankle sprain. The study sites include a public hospital and a private orthopedic clinic: the clinic has an orthopedic on-call service from Monday to Saturdays, and its vast facility includes an X-ray machine; and the hospital is a reference in highly-complex services and has an orthopedic on-call service 24 hours a day, 7 days a week.

The data collection instrument contained ten qualitative and quantitative variables: presence of ankle and/or foot fracture – the radiological finding; sex; age; skin color; history of ankle torsional trauma; affected side; time since injury; fracture site; surgery requirement; and the presence of at least one Ottawa criteria. Physicians available upon admission evaluated and treated all patients, with no interference from the researchers. The inclusion criteria were a history of ankle sprain and the request for ankle and/or foot radiographs, in addition to the examination. We excluded medical records without sufficient information for the OAR application. A radiologist analyzed all radiographs, and the report was attached to the medical records, enabling the retrospective analysis. A pediatric orthopedist monitored data collection and had the technical ability to interpret the radiographs without prior knowledge of the results or of the presence or absence of OARs in the patients' records. The image analysis by the radiologist and the pediatric orthopedist was fully concordant in terms of the identification of fractures. After the identification or not of the OARs in the patients' medical records and considering the agreement in the radiographic interpretations, we compared the radiographic findings with the OARs. As such, we calculated the clinical accuracy, sensitivity, specificity, positive predictive value (PPV), negative predictive value (NPV), and the positive and negative likelihood ratios of the OARs. To rule out fractures, a test must present high sensitivity, low negative likelihood ratio, and high NPV. This means that the test should yield few false negatives, being highly accurate in excluding fractures when the result is negative.

We analyzed the data collected using the IBM SPSS Statistics for Windows (IBM Corp., Armonk, NY, United States) software, version 21.0. The quantitative variables were expressed as mean and standard deviation values. The statistical tests considered a significance level of α = 0.05, indicating a 95% confidence level. The Kolmogorov-Smirnov test was used to verify the normality of the quantitative variables. Through the Pearson's Chi-squared test, followed by residual analysis when there was a statistical significance, we investigated the existence of associations involving the qualitative variables. We calculated the sensitivity, specificity, accuracy, PPV, NPV, and positive and negative likelihood ratios. The result of the radiological examination was considered the gold standard, and the presence of at least one Ottawa criterion, the diagnostic method being tested.

## Results

In total, 144 patients were eligible, and we excluded 47 patients from the sample due to the absence of torsional trauma or incomplete records, which prevented OAR application. Table 1 shows that the mean age of the sample was of 10.36 years, with a minimum age of 2 and a maximum of 16 years. Among the patients, 53.5% were male, and 98.6% were White.

**Table 1:** Characteristics of the sample

		95% confidence interval
	n = 144	
Age (years): mean ± standard deviation	10.36 ± 3.45 ^‡^	9.81–10.96
Sex: n (%)		
Male	77 (53.5)	–
Female	67 (46.5)	–
Skin color: n (%)		
White	142 (98.6)	–
Brown	1 (0.7)	–
Black	1 (0.7)	–

**Notes:**^‡^ Non-normal distribution per the Kolmogorov-Smirnov test ( *p* < 0.001); median (interquartile range): 11.0 (8.0–13.0).

Table 2 shows that 51.4% of the injuries were on the right side. Regarding the time of injury, 87.5% presented for consultation within ≤ 48 hours after the sprain. In addition, 20.1% of the patients had ankle and/or foot fractures according to the radiographic examination performed upon admission. There was a total of 35 fractures in 6 different regions: the distal tibia was the most common site (34.3%), followed by the fibula and lateral malleolus (with 22.9% each). Most patients (95.8%) underwent the conservative treatment.

**Table 2:** Clinical and diagnostic features and treatment of ankle lesions

	n = 144
Affected side: n (%)	
Right	74 (51.4)
Left	70 (48.6)
Lesion time: n (%)	
≤ 48 hours	126 (87.5)
> 48 hours	18 (12.5)
	
Presence of ankle and/or foot fractures (radiological result): n (%)	
Yes	29 (20.1)
No	115 (79.9)
Fracture site ( *n* = 29)* – n (%)	
Distal tibia	12 (34.3)
Fibula	8 (22.9)
Lateral malleolus	8 (22.9)
Base of the fifth metatarsal bone	4 (11.4)
Medial malleolus	2 (5.7)
Third metatarsal bone	1 (2.9)
Treatment: n (%)	
Conservative	138 (95.8)
Surgical	6 (4.2)

**Notes:** *Subjects can present fractures in more than one site.

Table 3 shows that 81 patients presented at least 1 Ottawa criterion in their first visit. Lateral malleolar pain and inability to walk four steps occurred in 35.4% and 34.7% of the patients, respectively. Among the additional criteria evaluated in patients with at least one positive criterion, 7.6% presented medial malleolar pain, 5.6%, pain at the base of the fifth metatarsal, and 2.1%, navicular pain.

**Table 3:** Ottawa protocol variables

	n = 144
Presence of at least one Ottawa criteria: n (%)	
Yes	81 (56.3)
No	63 (43.8)
	
Lateral malleolar pain on palpation: n (%)	
Yes	51 (35.4)
No	93 (64.3)
	
Medial malleolar pain on palpation: n (%)	
Yes	11 (7.6)
No	133 (92.4)
	
Inability to walk four steps: n (%)	
Yes	50 (34.7)
No	94 (65.3)
Pain at the base of the fifth metatarsal on palpation: n (%)	
Yes	8 (5.6)
No	136 (94.4)
Navicular pain on palpation: n (%)	
Yes	3 (2.1)
No	141 (97.9)

Table 4 describes the association between fractures and the Ottawa criteria. There was a statistically significant association between the presence of at least 1 OAR and fractures ( *p* < 0.001).

**Table 4:** Association between the presence of at least one Ottawa criteria and a fracture

	Fracture		*p* -value ^†^
	Yes	No	
	n = 29	n = 115	
Presence of at least one Ottawa criteria: n (%)			
Yes	29 (100.0) ^b^	52 (45.2)	< 0.001
No	0 (0.0)	63 (54.8) ^b^	

**Notes:**^†^ Value obtained after applying the Pearson's Chi-squared test; ^b^ tatistically significant value after the residual analysis ( *p* < 0.05).

Table 5 shows the distribution of the Ottawa criteria among the sample. The sensitivity and NPV of the rules in the sample were of 100.0% (sensitivity: 95% confidence interval [95%CI] = 96.2–100.0%; NPV: 95%CI = 95.7–100.0%) in detecting fractures, with a negative likelihood ratio of 0.00% (95% CI = 0.00–0.00%). The criteria for medial malleolar pain, pain at the base of the fifth metatarsal, and navicular pain showed high specificity in detecting fractures, reaching values greater than 90% in all cases. In addition, the criteria for lateral malleolar pain on palpation and inability to walk four steps presented NPVs of 91.4% and 94.7%, respectively.

**Table 5:** Distribution of the Ottawa criteria among the sample

	n = 144
Presence of at least one Ottawa criterion: % (95%CI)	
Sensitivity	100.0 (96.2–100.0)
Specificity	54.8 (47.3–62.0)
Accuracy	63.9 (55.7–72.0)
Positive predictive value	35.8 (27.5–46.5)
Negative predictive value	100.0 (95.7–100.0)
Positive likelihood ratio	2.21 (1.73–4.97)
Negative likelihood ratio	0.00 (0.00–0.00)
Medial malleolar pain on palpation: % (95%CI)	
Sensitivity	10.3 (0.0–21.4)
Specificity	93.0 (88.4–97.7)
Accuracy	76.4 (69.5–83.3)
Positive predictive value	27.3 (1.0–53.6)
Negative predictive value	80.5 (73.7–87.2)
Positive likelihood ratio	1.47 (0.00–9.30)
Negative likelihood ratio	0.96 (0.8–1.13)
Lateral malleolar pain on palpation: % (95%CI)	
Sensitivity	72.4 (56.1–88.7)
Specificity	73.9 (65.9–81.9)
Accuracy	73.6 (66.4–80.8)
Positive predictive value	41.2 (27.7–54.7)
Negative predictive value	91.4 (85.7–97.1)
Positive likelihood ratio	2.77 (1.65–4.90)
Negative likelihood ratio	0.37 (0.14–0.67)
	
Inability to walk four steps: % (95%CI)	
Sensitivity	82.8 (69.0–96.5)
Specificity	77.4 (69.7–85.0)
Accuracy	78.5 (71.8–85.2)
Positive predictive value	48.0 (34.2–61.8)
Negative predictive value	94.7 (90.1–99.2)
Positive likelihood ratio	3.66 (2.28–6.43)
Negative likelihood ratio	0.22 (0.04–0.44)
	
Pain at the base of the fifth metatarsal on palpation: % (95%CI)	
Sensitivity	17.2 (3.5–31.0)
Specificity	97.4 (94. –100.0)
Accuracy	81.3 (74.9–87.6)
Positive predictive value	62.5 (29.0–96.0)
Negative predictive value	82.4 (75.9–88.8)
Positive likelihood ratio	6.62 (0.64–310.0)
Negative likelihood ratio	0.85 (0.69–1.02)
	
Navicular pain on palpation: % (95%CI)	
Sensitivity	6.9 (0.0–16.1)
Specificity	99.1 (97.4–100.0)
Accuracy	80.6 (74.1–87.0)
Positive predictive value	66.7 (13.3–100.00)
Negative predictive value	80.9 (74.4–87.3)
Positive likelihood ratio	7.67 (0.00–161.0)
Negative likelihood ratio	0.94 (0.84–1.03)

**Abbreviation:** 95%CI, 95% confidence interval

Table 6 shows the relationship between the patients diagnosed with fractures through radiological examination and the presence of Ottawa criteria upon admission, revealing that 75.9% of the subjects presented 2 Ottawa criteria before radiography.

**Table 6:** Fracture description and presence of Ottawa criteria upon admission

	n = 29
One criterion: n (%)	5 (17.2)
Two criteria: n (%)	22 (75.9)
Three criteria: n (%)	2 (6.9%)

## Discussion

Torsional injuries are common in orthopedic emergency and urgent care services, resulting in fractures in 12% to 15% of cases. 11 In a meta-analysis of the OARs in 3,130 pediatric patients, Dowling et al. 12 identified fractures in 671 patients, with a prevalence of 21.4%. In the present study, the rate of fracture occurrence was similar, that is, 20.1% of the patients included, with 75.9% presenting 2 Ottawa criteria upon admission.

The most common childhood fracture occurs at the distal tibial physis, accounting for 9% to 18% of physeal injuries, surpassed only by fractures of the phalanges and distal radius. 14 In line with the literature, we observed that, of the 6 distinct fracture sites identified, the distal tibia region presented the highest frequency, with a rate of 34.3% of the fractures. The peak of these pediatric ankle fractures occurs from the ages of 8 to 15 years and the incidence rate is 2-fold higher in boys compared to girls. 2 Similarly, we observed that the average age for fracture presentation was of 10.36 years. In addition, there was a predominance (53.5%) of male subjects, which is consistent with the higher frequency in boys, although the incidence was not two-fold higher than in girls.

The initial management of these patients often involves routine foot and ankle radiographs. These are the second most frequently performed radiographs in the emergency department after spine radiographs. However, this cautious approach results in many unnecessary radiological examinations, leading to higher costs, longer treatment times, and avoidable radiation exposure. This led Stiell et al. 6 to develop and validate the OARs, which have become an extremely beneficial tool in managing ankle trauma, reducing the need for exam requests. 11

Yavas et al. 15 estimated a 28.4% reduction in radiograph requests by not performing them in patients with negative OARs. This results from the high sensitivity and moderate specificity of the method, which enables faster care and fewer exams. Similarly, in a meta-analysis including 15,581 patients from 27 studies, Bachmann et al. 10 confirmed that the OARs are a reliable clinical tool to exclude ankle and midfoot fractures. Furthermore, statistical calculations showed a possible reduction rate of 30% to 40% in unnecessary radiograph requests. 10 In the current study, we retrospectively observed that, if only patients with positive OARs underwent radiographic examinations, there would be a 43.8% (95%CI) reduction in the radiographs requested, which is in agreement with the available literature on the applicability of the test.

Plint et al. 13 found that the OARs reach 100% of sensitivity in detecting ankle and midfoot fractures in pediatric patients. The 667 patients studied by them 13 had 12 to 16 years of age and reported a previous episode of ankle trauma within 48 hours. The present study corroborates these findings, showing that 87.5% of patients sought care within 48 hours after the trauma and had similar minimum and maximum ages. Their statistical analysis showed the same sensitivity of 100.0%, NPV of 100.0%, and a negative likelihood ratio of 0.00%. Therefore, this demonstrates the greater probability of the child or adolescent not suffering a fracture if the AORs are negative, due to the high precision of the test in excluding fractures. The specificity levels were high for pain in the fifth metatarsal and on navicular palpation, with values of 97.4% and 99.1%, respectively. The NPV was high for pain on medial and lateral malleolus palpation, with values of 80.5% and 91.4%, respectively.

## Conclusion

The OARs appear to be a reliable clinical tool in the initial management of ankle sprains in pediatric patients, and they can be applied without concerns of missing significant fractures. We also observed a reduction in radiograph requests, which yields benefits such as reduced radiation exposure for children and adolescents, lower costs for the healthcare system, and shorter waiting times in the emergency department. Few international studies have analyzed the OARs in the pediatric population, and, to date, there are no Brazilian studies, reinforcing the need for further research to consolidate this clinical tool. Therefore, the results of the present study can serve as a basis for the formulation and implementation of government measures and guidelines aimed at improving the detection and treatment of ankle sprains.
