# Supplementary material for: Ottawa Ankle Rules: A Reliable Clinical Instrument to Detect Fractures in Children and Adolescents
Source: Rev Bras Ortop (Sao Paulo). 2025 Mar 4;60(1):s00441800938. doi: 10.1055/s-0044-1800938 (PMC11879485; doi:10.1055/s-0044-1800938)
Supplement: Related texts

# Research article: Regras do tornozelo de Ottawa: Um instrumento clínico confiável na detecção de fraturas na população infantojuvenil

**Keywords:** entorses e distensões, ferimentos e lesões, pé, pediatria, radiografia, tornozelo

## Abstract

**Objetivo** Avaliar a acurácia das regras do tornozelo de Ottawa (RTOs) aplicadas em prontuários de um hospital de alta complexidade e em uma clínica privada, assim como sua utilidade clínica em indivíduos com idade menor ou igual a 16 anos.

**Métodos** Trata-se de estudo observacional analítico transversal, com coleta de dados secundários de um departamento de emergência ortopédica e clínica ortopédica privada. A amostra foi representada por 144 pacientes, incluindo todos os pacientes com episódio torcional de tornozelo submetidos a radiografias de tornozelo e/ou pé quando da admissão. As RTOs foram adotadas como teste diagnóstico para o cálculo da sensibilidade, da especificidade, do valor preditivo positivo (VPP), do valor preditivo negativo (VPN) e das razões de verossimilhança positivas e negativas, e o resultado do exame radiográfico foi considerado o pardão-ouro.

**Resultados** Foram selecionados 191 pacientes, 144 dos quais foram incluídos após a aplicação dos critérios de inclusão. A sensibilidade e o VPN das RTOs na identificação de fraturas foram de 100%, sendo que o teste mais sensível e com o maior VPN foi a incapacidade de caminhar quatro passos. Identificou-se um potencial de redução de 43,8% no número total de radiografias solicitadas, caso apenas os pacientes com RTOs positivas fossem radiografados.

**Conclusões** As RTOs parecem ser um instrumento clínico confiável no manejo das entorses de tornozelo, e são um protocolo clínico útil na exclusão de fraturas em pacientes pediátricos. A utilização das regras evidenciou a sua capacidade de diminuir as solicitações desnecessárias de radiografias, o que minimiza a exposição à radiação e reduz os custos para o sistema de saúde.

## Introdução

As lesões de tornozelo são comuns nos departamentos de emergência, e representam de 6 a 12% dos atendimentos. 1 Destas, as fraturas de tornozelo configuram aproximadamente 5% de todas as fraturas em pacientes pediátricos. 2 Grande parte dessas lesões apresentam pico de incidência que varia entre 8 e 15 anos de idade, além de serem 2 vezes mais frequentes em meninos. 3 A maioria dos pacientes com traumas no pé e tornozelo são submetidos a radiografias no pronto atendimento devido às chances de eventuais fraturas, embora sua frequência seja detectada em menos de 15% dos casos. Essa abordagem conservadora gera muitas solicitações desnecessárias de radiografias, o que acarreta exposição dispensável à radiação, custos adicionais para o sistema de saúde e tempo de espera prolongado para os pacientes. 4

Para padronizar os métodos diagnósticos e diminuir as solicitações dispensáveis de radiografias, um grupo de pesquisadores formulou, em 1992, as regras do tornozelo de Ottawa (RTOs, que permitem prever a ocorrência de fraturas em lesões agudas do tornozelo mediante de um exame físico direcionado. 5 O protocolo afirma que a avaliação radiográfica só é essencial quando o paciente relatar dor maleolar associada a dor maleolar lateral ou medial à palpação óssea (ambas 6 cm distais na região posterior) ou incapacidade de apoiar o membro (imediatamente após a lesão e ao caminhar 4 passos na urgência). Outra indicação seria em casos de trauma no tornozelo com dor referida no mediopé associada a um ou mais dos seguintes critérios: dor à palpação óssea da base do quinto metatarso, dor à palpação óssea do navicular e incapacidade de apoiar o membro (imediatamente após a lesão e ao caminhar quatro passos na urgência). 6 A princípio somente a radiografia é necessária quando os critérios clínicos são atendidos. 7 Os demais exames complementares, como tomografia computadorizada (TC), ressonância magnética (RM) e exames de sangue, são reservados para situações específicas, como em avaliações de congruência articular, lesões intra-articulares e planejamento cirúrgico. 8 O protocolo é contraindicado em pacientes com estado mental alterado, intoxicação por drogas, barreiras de linguagem ou edema significativo que limita a palpação óssea. 6

A sensibilidade das regras atinge valores de quase 100% em estudos que envolvem população adulta. 9 Entretanto, autores internacionais 10 11 12 13 identificaram uma oportunidade de utilizar as RTOs na população pediátrica. Essa aplicação na área pediátrica é pouco utilizada, possivelmente devido à maior dificuldade de se obter um histórico verbal confiável e realizar um exame físico apropriado em algumas crianças, quando comparadas aos adultos. Além disso, as RTOs só podem ser aplicadas em crianças capazes de caminhar independentemente antes do episódio torcional. 12

As RTOs já foram validadas em adultos, mas carecem de estudos com a população infantojuvenil para sua possível utilização como instrumento de triagem diagnóstica. No presente estudo, temos o propósito de avaliar sua aplicabilidade em pacientes pediátricos atendidos em um hospital de alta complexidade e em uma clínica privada.

## Materiais e Métodos

Realizou-se um estudo observacional, analítico e transversal com abordagem quantitativa. O projeto foi aprovado e supervisionado pelo Comitê de Ética da instituição (CAAE: 60851622.1.0000.0119).

A amostra incluiu 144 pacientes com idade menor ou igual a 16 anos, atendidos entre janeiro de 2019 e dezembro de 2022, e que realizaram radiografias após episódio de entorse de tornozelo. Os locais de estudo abrangem um hospital público e uma clínica ortopédica privada: a clínica conta com plantão ortopédico de segunda-feira a sábado, e oferece uma ampla estrutura, com raio-X à disposição; já o hospital é referência em serviços de alta complexidade e conta com plantão ortopédico 24 horas por dia, 7 dias por semana.

O instrumento de coleta de dados continha dez variáveis qualitativas e quantitativas: presença de fratura no tornozelo e/ou pé – resultado do exame de raio x; sexo; idade; cor; histórico de trauma torcional de tornozelo; lado acometido; tempo de lesão; local da fratura; necessidade de cirurgia; e presença de pelo menos um dos critérios de Ottawa. Todos os pacientes foram avaliados e tratados pelos médicos disponíveis na admissão, sem interferência dos pesquisadores. Os pacientes foram incluídos no estudo caso houve histórico de entorse de tornozelo e solicitação de radiografias de tornozelo e/ou pé, além da realização do exame. Os prontuários sem informações suficientes para a aplicação das RTOs foram excluídos. Todas as radiografias foram analisadas por um médico radiologista, e os resultados foram anexados aos prontuários, o que permitiu a análise retrospectiva. Um ortopedista pediátrico acompanhou a coleta de dados e tinha capacidade técnica para interpretar as radiografias, sem conhecimento prévio dos resultados ou da presença ou ausência das RTOs nos registros dos pacientes. A análise das imagens pelo radiologista e pelo ortopedista pediátrico foi totalmente concordante quanto à identificação de fraturas. Após a identificação ou não das regras nos prontuários dos pacientes e considerando a concordância das interpretações radiográficas, realizou-se uma comparação entre os resultados radiográficos e as RTOs. Com isso, pôde-se calcular a acurácia clínica, a sensibilidade, a especificidade, o valor preditivo positivo (VPP), o valor preditivo negativo (VPN), bem como as razões de verossimilhança positivas e negativas das regras. Para descartar fraturas, é crucial que o teste tenha alta sensibilidade, baixa razão de verossimilhança negativa e alto VPN; isso significa que o teste deve render poucos falsos negativos e ser altamente preciso na exclusão de fraturas quando o resultado é negativo.

Os dados coletados foram analisados por meio do programa IBM SPSS Statistics for Windows (IBM Corp., Armonk, NY, United States), versão 21.0. As variáveis quantitativas foram expressas em valores de média e desvio padrão. Os testes estatísticos foram realizados com nível de significância α = 0,05, indicando confiança de 95%. A normalidade das variáveis quantitativas foi verificada pelo teste de Kolmogorov-Smirnov. A investigação da existência de associações entre as variáveis qualitativas foi realizada com a aplicação do teste do Qui-quadrado de Pearson, seguida de análise de resíduo quando observada significância estatística. Foram calculadas a sensibilidade, a especificidade, a acurácia, o VPP e o VPN, além das razões de verossimilhança positivas e negativas. O padrão-ouro considerado foi o resultado do exame de raio X e, como método diagnóstico em teste, a presença de pelo menos um dos critérios de Ottawa.

## Resultados

No total, 144 pacientes foram caracterizados como elegíveis, e 47 pacientes foram excluídos da amostra devido à ausência de trauma torcional ou por registros incompletos, o que impedia a aplicação das RTOs. A Tabela 1 mostra que a média de idade da amostra foi de 10,36 anos, com idade mínima de 2 anos e máxima 16 anos. Entre os pacientes, 53,5% eram do sexo masculino, e 98,6% eram brancos.

**Tabela 1:** Características da amostra

		Intervalo de confiança de 95%
	n = 144	
Idade (anos): média ± desvio padrão	10,36 ± 3,45 ^‡^	9,81–10,96
Sexo: n (%)		
Masculino	77 (53,5)	–
Feminino	67 (46,5)	–
Cor da pele: n (%)		
Branca	142 (98,6)	–
Parda	1 (0,7)	–
Preta	1 (0,7)	–

**Notas:**^‡^ Distribuição não normal após a aplicação do teste de Kolmogorov-Smirnov ( *p* < 0,001); mediana (intervalo interquartílico): 11,0 (8,0–13,0).

Nota-se, na Tabela 2 , que 51,4% das lesões foram no lado direito. Em relação ao tempo de lesão, 87,5% se apresentaram para consulta em um período ≤ 48 horas após a entorse. Observou-se também que 20,1% dos pacientes apresentaram fratura no tornozelo e/ou pé conforme o exame radiográfico realizado à admissão. Foram registradas 35 fraturas em 6 regiões distintas: a região da tíbia distal foi a mais comum (34,3%), seguida das regiões da fíbula e do maléolo lateral, ambas com 22,9%. Em 95,8% dos pacientes, optou-se pelo tratamento conservador.

**Tabela 2:** Características clínicas, diagnósticas e tratamento das lesões de tornozelo

	n = 144
Lado acometido: n (%)	
Direito	74 (51,4)
Esquerdo	70 (48,6)
Tempo de lesão: n (%)	
≤ 48 horas	126 (87,5)
> 48 horas	18 (12,5)
Presença de fratura no tornozelo e/ou pé (resultado da radiografia) : n (%)	
Sim	29 (20,1)
Não	115 (79,9)
Local da fratura ( *n* = 29)* – n (%)	
Tíbia distal	12 (34,3)
Fíbula	8 (22,9)
Maléolo lateral	8 (22,9)
Base do quinto metatarso	4 (11,4)
Maléolo medial	2 (5,7)
Terceiro metatarso	1 (2,9)
Tratamento: n (%)	
Conservador	138 (95,8)
Cirúrgico	6 (4,2)

**Nota:** *Indivíduos podem apresentar fratura em mais de um local.

Na Tabela 3 , observa-se que 81 pacientes apresentaram pelo menos 1 critério de Ottawa em seu primeiro atendimento. A dor maleolar lateral e a incapacidade de deambulação por 4 passos foram observadas em 35,4% e 34,7% dos pacientes, respectivamente. Entre os critérios adicionais avaliados nos pacientes com pelo menos 1 critério positivo, constata-se que 7,6% apresentaram dor maleolar medial, 5,6%, dor na base do quinto metatarso, e 2,1%, dor navicular.

**Tabela 3:** Descrição das variáveis do protocolo de Ottawa

	n = 144
Presença de pelo menos um dos critérios de Ottawa: n (%)	
Sim	81 (56,3)
Não	63 (43,8)
Dor maleolar lateral à palpação: n (%)	
Sim	51 (35,4)
Não	93 (64,3)
Dor maleolar medial à palpação: n (%)	
Sim	11 (7,6)
Não	133 (92,4)
Incapacidade de deambular por quatro passos: n (%)	
Sim	50 (34,7)
Não	94 (65,3)
Dor à palpação na base do quinto metatarso: n (%)	
Sim	8 (5,6)
Não	136 (94,4)
Dor navicular à palpação: n (%)	
Sim	3 (2,1)
Não	141 (97,9)

A Tabela 4 descreve a associação entre a presença de fratura e os critérios de Ottawa. Observa-se associação estatisticamente significativa entre a presença de pelo menos 1 das RTOs e a presença de fratura ( *p* < 0,001).

**Tabela 4:** Associação entre a presença de pelo menos um dos critérios de Ottawa e presença de fratura

	Presença de fratura		Valor de *p* ^†^
	Sim	Não	
	n = 29	n = 115	
Presença de pelo menos um dos critérios de Ottawa: n (%)			
Sim	29 (100,0) ^b^	52 (45,2)	< 0,001
Não	0 (0,0)	63 (54,8) ^b^	

**Notas:**^†^ Valor obtido após a aplicação do teste do Qui-quadrado de Pearson; ^b^ valor estatisticamente significativo após análise de resíduo ( *p* < 0,05).

A distribuição dos critérios de Ottawa na amostra foi abordada na Tabela 5 . A sensibilidade e o VPN das regras em nossa amostra foram de 100,0% (sensibilidade: intervalo de confiança de 95% [IC95%] = 96,2–100,0%; VPN: IC95% = 95,7–100,0%) na detecção de fraturas, com uma razão de verossimilhança negativa de 0,00% (IC95% = 0,00–0,00%). Os critérios de dor maleolar medial, dor na base do quinto metatarso e dor navicular mostraram alta especificidade na detecção de fraturas, e atingiram valores acima de 90% em todos os casos. Além disso, os critérios de dor maleolar lateral à palpação e incapacidade de deambular por 4 passos apresentaram VPN de 91,4% e 94,7%, respectivamente.

**Tabela 5:** Distribuição dos critérios de Ottawa na amostra

	n = 144
Presença de pelo menos um dos critérios de Ottawa: % (IC95%)	
Sensibilidade	100,0 (96,2–100,0)
Especificidade	54,8 (47,3–62,0)
Acurácia	63,9 (55,7–72,0)
Valor preditivo positivo	35,8 (27,5–46,5)
Valor preditivo negativo	100,0 (95,7–100,0)
Razão de verossimilhança positiva	2,21 (1,73–4,97)
Razão de verossimilhança negativa	0,00 (0,00–0,00)
Dor maleolar medial à palpação: % (IC95%)	
Sensibilidade	10,3 (0,0–21,4)
Especificidade	93,0 (88,4–97,7)
Acurácia	76,4 (69,5–83,3)
Valor preditivo positivo	27,3 (1,0–53,6)
Valor preditivo negativo	80,5 (73,7–87,2)
Razão de verossimilhança positiva	1,47 (0,00–9,30)
Razão de verossimilhança negativa	0,96 (0,8–1,13)
Dor maleolar lateral à palpação: % (IC95%)	
Sensibilidade	72,4 (56,1–88,7)
Especificidade	73,9 (65,9–81,9)
Acurácia	73,6 (66,4–80,8)
Valor preditivo positivo	41,2 (27,7–54,7)
Valor preditivo negativo	91,4 (85,7–97,1)
Razão de verossimilhança positiva	2,77 (1,65–4,90)
Razão de verossimilhança negativa	0,37 (0,14–0,67)
	
Incapacidade de deambular por quatro passos: % (IC95%)	
Sensibilidade	82,8 (69,0–96,5)
Especificidade	77,4 (69,7–85,0)
Acurácia: % (IC95%)	78,5 (71,8– 5,2)
Valor preditivo positivo	48,0 (34,2–61,8)
Valor preditivo negativo	94,7 (90,1–99,2)
Razão de verossimilhança positiva	3,66 (2,28–6,43)
Razão de verossimilhança negativa	0,22 (0,04–0,44)
	
Dor base quinto metatarso à palpação: % (IC95%)	
Sensibilidade	17,2 (3,5–31,0)
Especificidade	97,4 (94,5–100,0)
Acurácia	81,3 (74,9–87,6)
Valor preditivo positivo	62,5 (29,0–96,0)
Valor preditivo negativo	82,4 (75,9–88,8)
Razão de verossimilhança positiva	6,62 (0,64–310,0)
Razão de verossimilhança negativa	0,85 (0,69–1,02)
	
Dor navicular à palpação: % (IC95%)	
Sensibilidade	6,9 (0,0–16,1)
Especificidade	99,1 (97,4–100,0)
Acurácia	80,6 (74,1–87,0)
Valor preditivo positivo	66,7 (13,3–100,00)
Valor preditivo negativo	80,9 (74,4–87,3)
Razão de verossimilhança positiva	7,67 (0,00–161,0)
Razão de verossimilhança negativa	0,94 (0,84–1,03)

Abreviatura: IC95%, intervalo de confiança de 95%.

A Tabela 6 apresenta a relação entre os pacientes da amostra que foram diagnosticados com fratura por meio do exame radiológico e a presença dos critérios de Ottawa à admissão. Verifica-se que 75,9% dos pacientes apresentaram 2 critérios de Ottawa antes da realização da radiografia.

**Tabela 6:** Descrição da presença de fratura e da presença dos critérios de Ottawa à admissão para o atendimento

	n = 29
Um critério: n (%)	5 (17,2)
Dois critérios: n (%)	22 (75,9)
Três critérios: n (%)	2 (6,9%)

## Discussão

Lesões torcionais são comuns nos serviços de urgência e emergência ortopédicos, e cursam com fraturas em 12% a 15% dos casos. 11 Em uma metanálise das RTO em 3.130 pacientes pediátricos, Dowling et al. 12 identificaram a presença de fraturas em 671 pacientes da amostra, sendo constatada uma prevalência de 21,4%. Neste estudo, a taxa de ocorrência de fraturas foi semelhante, e foi observada em 20,1% dos pacientes incluídos, sendo que 75,9% desses pacientes apresentaram 2 critérios de Ottawa à admissão.

A fratura infantil mais comum é a da fise distal da tíbia, que representa de 9% a 18% das lesões fisárias, e é superada apenas pelas fraturas de falanges e do rádio distal. 14 Em concordância com a literatura, observou-se neste estudo que, dos 6 locais distintos de fraturas identificados, a região da tíbia distal apresentou a maior frequência com uma taxa de 34,3% das fraturas. É sabido que essas fraturas pediátricas de tornozelo atingem seu pico entre 8 e 15 anos de idade, com uma taxa de incidência dobrada em meninos quando comparada às meninas. 2 De modo semelhante, observamos neste estudo que a idade média de apresentação das fraturas foi de 10,36 anos. Além disso, houve predominância (53,5%) do sexo masculino, o que também corrobora a ocorrência mais frequente de fraturas em meninos, embora não apresente uma incidência duplicada.

O manejo inicial desses pacientes frequentemente envolve a realização de radiografias de rotina de pé e tornozelo, sendo o segundo tipo de radiografia mais realizado no pronto-socorro, logo após as radiografias de coluna. Entretanto, essa abordagem cautelosa resulta em muitos exames radiológicos desnecessários, o que leva a custos mais altos, maior tempo de atendimento e exposição desnecessária à radiação. Devido a isso, Stiell et al. 6 desenvolveram e validaram as RTO, tornando-se uma ferramenta extremamente benéfica no atendimento dos traumas de tornozelo, reduzindo a necessidade de solicitações do exame. 11

Yavas et al. 15 estimaram uma redução de 28,4% na solicitação de radiografias caso elas não fossem realizadas em pacientes com RTOs negativas. Isso está relacionado aos altos valores de sensibilidade e à especificidade moderada do método, que permite agilizar o atendimento e diminuir a realização do exame. Da mesma forma, a metanálise conduzida por Bachmann et al., 10 que incluiu 15.581 pacientes de 27 estudos, confirmou que as RTOs são um instrumento clínico confiável para exclusão de fraturas no tornozelo e no mediopé. Além disso, os cálculos estatísticos evidenciaram uma possível taxa de redução de 30% a 40% na solicitação de radiografias desnecessárias. 10 No presente estudo, observou-se retrospectivamente que, caso somente pacientes com RTOs positivas realizassem exames radiográficos, haveria uma redução de 43,8% (IC95%) nas radiografias solicitadas, oi que está em concordância com a literatura disponível sobre a aplicabilidade do teste.

Plint et al. 13 detectaram que as RTOs atingem 100% de sensibilidade na detecção de fraturas no tornozelo e no mediopé em pacientes pediátricos. Os 667 pacientes estudados por eles 13 tinham entre 12 e 16 anos de idade e relataram episódio prévio de trauma em tornozelo dentro de 48 horas. Este estudo corrobora esses achados, pois mostra que 87,5% dos pacientes buscaram atendimento dentro de 48 horas após o trauma e tinham idades mínima e máxima similares. Na análise estatística, foi observada a mesma sensibilidade de 100,0%, VPN de 100,0% e razão de verossimilhança negativa de 0,00%. Isso demonstra, portanto, a maior probabilidade de a criança ou o adolescente não apresentarem fratura perante o resultado negativo das RTOs devido à alta precisão do teste na exclusão de fraturas. Altos níveis de especificidade foram observados quanto aos critérios de dor à palpação do quinto metatarso e dor navicular, sendo encontrados valores de 97,4% e 99,1%, respectivamente. Observou-se também alto VPN nos critérios de dor à palpação maleolar medial e lateral, sendo encontrados valores de 80,5% e 91,4%, respectivamente.

## Conclusão

As RTOs parecem ser um instrumento clínico confiável no manejo inicial de entorses do tornozelo em pacientes pediátricos, que pode ser aplicado sem a preocupação de perder fraturas significativas. Observou-se também uma redução na solicitação de radiografias, o que traz benefícios como menor exposição à radiação para crianças e adolescentes, redução de custos para o sistema de saúde e menor tempo de espera no setor de emergência. Há uma escassez de pesquisas internacionais sobre as RTOs na população pediátrica, e, até o momento, nenhum estudo publicado no Brasil, o que reforça a necessidade de novas pesquisas para a consolidação desse instrumento clínico. Portanto, os resultados deste estudo podem servir como base para a formulação e a implementação de medidas governamentais e de diretrizes que visem melhorar a detecção e o tratamento de entorses no tornozelo.
